# Comorbid Insomnia and Sleep Apnea Across the Pediatric Age: A Polysomnographic Study

**DOI:** 10.3390/children12091250

**Published:** 2025-09-17

**Authors:** Lisa Brunel, Marion Comajuan, Sabine Plancoulaine, Benjamin Putois, Julien Lioret, Marine Thieux, Laurianne Coutier, Patricia Franco, Aurore Guyon

**Affiliations:** 1 INSERM U1028/CNRS UMR 5292, Lyon Neuroscience Research Center (CRNL), University Lyon 1, 69500 Bron, France; ext-lisa.brunel@chu-lyon.fr (L.B.); marion.comajuan@chu-lyon.fr (M.C.); sabine.plancoulaine@inserm.fr (S.P.); benjamin.putois@unidistance.ch (B.P.); ext-julien.lioret@chu-lyon.fr (J.L.); marine.thieux@univ-lyon1.fr (M.T.); laurianne.coutier@chu-lyon.fr (L.C.); aurore.guyon@chu-lyon.fr (A.G.); 2 Pediatric Sleep Unit, Hôpital Femme-Mère Enfant, Hospices Civils de Lyon, 69500 Bron, France; 3 Faculty of Psychology, Swiss Distance Learning University, 3900 Brig, Switzerland; 4 Service de Pneumologie Infantile, Allergologie et Centre de Référence en Mucoviscidose, Hôpital Femme Mère Enfant, Hospices Civils de Lyon, 69500 Bron, France

**Keywords:** pediatrics, polysomnography, obstructive sleep apnea, COMISA, sleep, anxiety

## Abstract

**Highlights:**

**What are the main findings?**

- Comorbid insomnia and sleep apnea (COMISA) is less common in children than in adults.
- Among patients with obstructive sleep apnea (OSA), those with comorbid insomnia demonstrated a lower OSA severity but higher anxiety levels.

**What is the implication of the main finding?**

- The mechanisms underlying COMISA may differ between children and adults, highlighting the need for age-specific approaches to diagnosis and treatment.
- Anxiety should systematically be assessed in children with OSA, as it may be a risk factor for COMISA.

**Abstract:**

Background/Objectives: Comorbid insomnia and sleep apnea (COMISA) in children is poorly documented. This study aimed to evaluate the frequency of COMISA and to explore its clinical and polysomnographic characteristics in children referred for polysomnography (PSG) for any sleep complaint. Methods: All patients with a complete insomnia sub-score on the Sleep Disturbance Scale for Children (SDSC; for children from 6 months to 16 years old) who underwent a night PSG in a pediatric sleep unit (2018–2024) were included in this retrospective study. Pathological SDSC insomnia sub-score defined insomnia and obstructive apnea-hypopnea index ≥ 2/h on PSG defined OSA. Questionnaires regarding sleepiness, depression, anxiety, and hyperactivity were also collected. Results: Children had isolated insomnia in 11.5% of cases, isolated OSA in 37.5%, and COMISA in 13.5%. Insomnia frequency was not different between patients with and those without OSA (26.5% vs. 23.5%). COMISA was more frequent in patients under 4 years old than in older ones (39.1% vs. 5.8%). No polysomnographic or clinical characteristic of COMISA was identified, except that OAHI was higher in children with isolated OSA. Patients with COMISA or isolated insomnia were more anxious than those with isolated OSA. Conclusions: Unlike in adults, the present findings do not support a mutual association between OSA and insomnia in children. OSA severity was lower in children with COMISA. Anxiety levels were higher in children with insomnia, regardless of the presence of OSA, suggesting that anxiety should be assessed in all children with OSA.

## 1. Introduction

Obstructive sleep apnea (OSA) affects 1–5% of the pediatric population worldwide [1]. It is associated with decreased school performance, impaired memory and attention, hyperactivity, sleepiness, and cardiovascular repercussions such as hypertension [1].

The frequency of insomnia in childhood varies according to age, from 20–30% in infants and preschoolers to 11% in adolescents [2]. Until five years old, insomnia is mostly behavioral; in adolescence, however, it is associated with phase delay, poor sleep hygiene, and psychological causes [2]. Chronic insomnia has negative repercussions on behavior, mood, and cognition [2,3,4,5].

The co-occurrence of OSA and insomnia is known as comorbid insomnia and sleep apnea (COMISA). In adults, the prevalence of insomnia reaches 39–55% in patients with OSA vs. 10% in the general population [6]. COMISA is associated with more deleterious impacts than either isolated disorder: greater sleep impairments, reduced quality of life [6], higher rates of psychiatric disorders, more anxiety, cognitive complaints, irritability [7], and higher risks of cardiovascular comorbidities [8]. In addition, insomnia can lower adherence to OSA treatment [6].

The characteristics of COMISA in children remain poorly defined. To date, only two retrospective studies have addressed this topic [9,10]; however, they involved heterogeneous populations in terms of age and sleep complaints and employed different methods to assess insomnia.

This study aimed to (1) determine the frequency of COMISA in children aged 6 months to 16 years referred for polysomnography (PSG) in a sleep clinic, and (2) explore the demographic (age, sex, body mass index—BMI, etc.), polysomnographic (i.e., sleep macro-architecture, respiratory parameters), and clinical characteristics (i.e., sleepiness, anxiety, depressive symptoms, hyperactivity, school difficulties) of children with COMISA compared to those with isolated OSA or insomnia. We hypothesized a high prevalence of insomnia among children with OSA, and we anticipated that those with COMISA would exhibit more severe sleep disturbance and daytime impairments, consistent with findings in adults.

## 2. Materials and Methods

### 2.1. Patients

This retrospective cross-sectional study included patients aged from 6 months to 16 years who underwent overnight PSG without respiratory support at the pediatric sleep unit of the Hospices Civils de Lyon (HCL, Lyon, France) between February 2018 and March 2024, irrespective of referral indication. Inclusion required complete caregiver responses to the Disorders of Initiating and Maintaining Sleep (DIMS) subscale of the Sleep Disturbance Scale for Children (SDSC), which is validated for this age range.

### 2.2. Ethical Considerations

The study was approved by the institutional ethics committee of the HCL (n°24_149, 23 July 2024) and declared to the French national data protection authority (Commission Nationale de l’Informatique et des Libertés, CNIL, n°24-5149). Patients and caregivers were informed about the use of clinical data for research purposes.

### 2.3. Demographic Characteristics

Age, sex, body mass index (BMI), and medical history were collected from medical records. BMI z-score was computed as a measure of weight adjusted for height, sex, and age relative to a smoothed reference distribution [11]. Obesity was determined in children aged ≥2 years using the International Obesity Task Force curves of BMI [12]. Preterm birth (gestational age < 37 weeks), neurodevelopmental disorders, and the use of medications with wake-promoting or hypnotic effects were noted, as these factors may influence OSA and insomnia. Data on attention deficit hyperactivity disorder (ADHD) were not available for children aged under 4 years.

### 2.4. Polysomnography

Overnight PSG included 8 electroencephalogram (EEG) electrodes referenced to the mastoids according to the 10–20 system, right and left electrooculograms, electromyography on the levator menti surface and left and right anterior tibialis muscle (for patients ≥ 2 years old), thoraco-abdominal belts, nasal cannula, oro-nasal thermistor, oximetry for oxygen saturation (SpO_2_), and electrocardiogram. Sampling frequency was 256 Hz; impedances were kept below 10 kΩ. Sleep was scored visually by experienced specialists.

#### 2.4.1. Sleep Architecture

The following sleep architecture parameters were calculated based on the AASM 2007 guidelines [13]: total sleep time (TST in minutes, min), sleep efficacy (SE, %; ratio of TST on total sleep period), sleep onset latency (SOL, min), wake after sleep onset (WASO, min), percentage of stages N1, N2, N3, and rapid eye movement (REM) sleep relative to TST, arousal index (/h), and arousal-and-awakening index (/h). Periodic leg movement index (PLMI, /h) was recorded for children aged 2 years and older. A SOL of 0 min was considered indicative of a recording initiated after sleep onset; such cases were excluded from the analyses (*n* = 3).

#### 2.4.2. Respiratory Parameters

The following respiratory parameters were calculated based on the AASM 2012 pediatric guidelines [14] according to TST: obstructive apnea-hypopnea index (OAHI, /h; including obstructive apneas and hypopneas, and mixed apneas), central apnea-hypopnea index (CAHI/h), respiratory effort-related arousal (RERA) index (/h), mean CO_2_ (mmHg), time spent with CO_2_ >50 mmHg (CO_2_ > 50 mmHg, %), oxygen desaturation index >3% (ODI > 3, /h), mean SpO_2_ (%), and time spent with SpO_2_ < 90% (min). Patients with CAHI ≥ 5/h were excluded (*n* = 8).

### 2.5. Questionnaires

The SDSC-DIMS score was used to evaluate insomnia. Two age-specific versions exist, for children aged 6 months to 4 years [15] (8 items; total score range: 2–40) and for those aged 4–16 years [16] (7 items; total score range: 7–35).

An adapted version of the Epworth Sleepiness Scale for children and adolescents [17], validated in French as the FSSA [18], was used to assess daytime sleepiness in children aged 6 years and older. Scores above 10 were considered indicative of excessive daytime sleepiness.

Anxiety was evaluated through the Child Behavior Checklist (CBCL) [19,20]. The validated French version of this questionnaire assessing behavioral and emotional problems was answered by parents of children aged 4 years and older [21]. For this study, the anxiety/depression subscale was collected. Normalized scores ≥ 70 were considered clinically significant.

The Children’s Depression Inventory (CDI) was used to evaluate depressive symptoms in children above 7 years old [22]. Scores ≥ 16 were considered as indicative of clinically significant depressive feelings.

Hyperactivity was assessed in children aged 6 years and older using the Conners Abbreviated Rating Scale [23]. Scores > 15 were considered clinically relevant.

Information on school difficulties was collected from medical reports and by referring to item 61 of the CBCL (“poor school performance”). Patients under 4 years old, those not attending school, and those with missing schooling status data were excluded from the analysis for this variable.

### 2.6. Definition of Obstructive Sleep Apnea and Insomnia

OSA was defined as an OAHI ≥ 2/h [24,25] and classified as mild (OAHI < 5/h), moderate (OAHI ≥ 5/h and <10/h), or severe (OAHI ≥ 10/h). OSA type was based on comorbidities: type III for craniofacial, neuromuscular, or syndromic comorbidities; type II in case of obesity; and type I in the absence of such comorbidities [26].

Insomnia was defined as SDSC-DIMS score >16/40 for children aged under 4 years and >21/35 for children aged 4 years and older. Additional sub-scores were computed to assess specific components: difficulties initiating sleep (by adding the scores of the items “sleep onset latency”, “reluctant to go to bed”, “difficulties falling asleep”, and “anxiety/fear when going to sleep”) and difficulties maintaining sleep (score on the item “≥2 awakenings per night”).

### 2.7. Statistical Analyses

Two age groups were defined based on the SDSC versions: a “younger group” (<4 years old) and an “older group” (≥4 years old). Patients were further categorized into 4 groups according to their OSA and insomnia status: no OSA or insomnia (“no-OSA no-INS”), isolated OSA (“OSA-only”), isolated insomnia (“INS-only”), and COMISA.

Comparisons were conducted by age group and according to OSA/insomnia status. In the younger group, sample size allowed comparisons only between OSA-only and COMISA groups. All 4 groups were compared within the older group.

Statistical analyses were conducted using Python (version 3.11.7) [27] with the SciPy [28] and Pingouin [29] packages. The normality of data distributions was assessed using the Shapiro–Wilk test. As most continuous variables were not normally distributed, non-parametric tests were performed: Kruskal–Wallis (≥3 groups) or Mann–Whitney (2 groups and post hoc 2-by-2 comparisons) for continuous variables, and *χ*^2^ or Fisher’s exact test for discrete variables. Patients with missing data for a given variable were excluded from the analysis of that variable.

Continuous variables are expressed as median [range] and discrete variables as count (%). The absolute value of Mann–Whitney r is reported to evaluate effect size for continuous variables; φ and ω are reported respectively for dichotomous and polytomous discrete variables. Effect size was considered small (<0.3), medium (0.3–0.5), or large (≥0.5). Significance level was set at 0.05. The Benjamini–Hochberg method was applied to control for the false discovery rate (FDR) [30].

## 3. Results

### 3.1. Patient Characteristics

A total of 200 patients were included (47.5% females; age = 9.1 years [0.6–16.7]). Table 1 summarizes patient characteristics per age group.

#### 3.1.1. Demographic Characteristics

Proportions of female sex, preterm birth, and obesity did not differ according to age group. In the older group, neurodevelopmental disorders and wake-promoting medications were more frequent.

The primary indication for PSG in both age groups was suspicion or screening of sleep disordered breathing (SDB), with a higher proportion observed in the younger group. In contrast, PSG was more frequently motivated by complaints of insomnia or hypersomnia in the older than in the younger group. Among patients with suspected SDB, the distribution of OSA types (type I/II/III) was similar between age groups (younger group 51.2%/0%/48.8%; older group 46.6%/9.1%/44.3%; *p* = 0.1).

#### 3.1.2. OSA and Insomnia

OAHI was significantly higher and OSA more frequent in the younger group. However, the distribution of OSA types and severity levels was similar between the two age groups.

**Figure 1:**
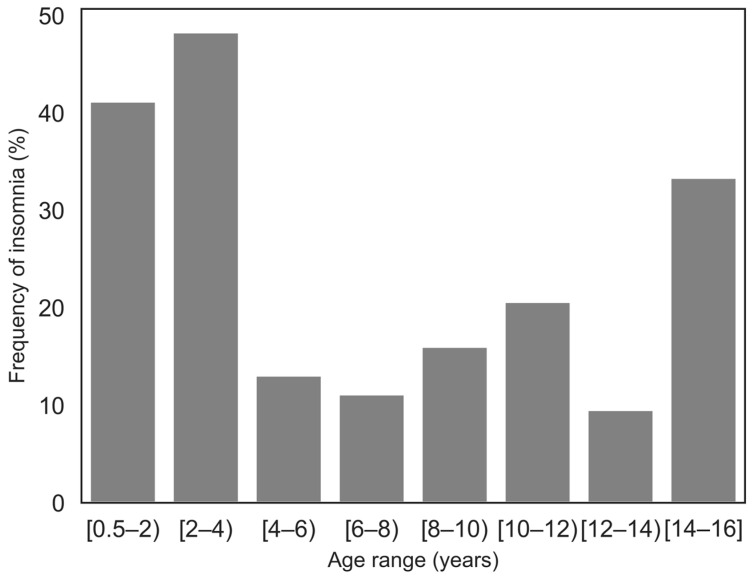
Frequency of insomnia by age range.

Insomnia was more frequent in the younger group. Figure 1 shows the frequency of insomnia across finer age subgroups. Difficulties initiating sleep tended to be more pronounced in the older group, while the severity of difficulties maintaining sleep was comparable between age groups.

In the overall cohort, COMISA was identified in 27 patients (13.5%). Among those with OSA, the frequency of COMISA was 26.5%, with a higher proportion observed in the younger group. Insomnia was present in 23 patients (23.5%) without OSA, and its frequency did not differ significantly between those with and those without OSA (younger group *p* = 0.3; older group *p* = 0.2).

### 3.2. Characteristics Associated with COMISA

Patients’ characteristics were compared according to the presence/absence of OSA and/or insomnia, separately within each age group. These comparisons are summarized in Table 2 for the younger group and Table 3 for the older group.

#### 3.2.1. Demographic Characteristics

Age, sex, and BMI were similar across the groups. The prevalence of neurodevelopmental disorders and the use of medications potentially affecting sleep did not differ significantly between the COMISA group and the other groups. In the older group, ADHD was more frequently diagnosed in patients with INS-only compared to those with isolated OSA; however, ADHD prevalence in the COMISA group did not differ significantly from either of the other groups.

In the younger group, all patients in the OSA-only group were referred for PSG due to suspicion or screening of SDB. In contrast, patients with COMISA were referred for SDB (83.3%), insomnia complaints (11.1%), or parasomnia (5.6%). The difference in referral indications between the two groups was not statistically significant (*p* = 0.2).

In the older group, patients in the INS-only group were referred for PSG due to insomnia (65.0%), suspected SDB (30.0%), or hypersomnia complaints (5.0%). Patients with OSA-only were referred primarily for suspected SDB (73.7%), followed by hypersomnia (15.8%) and insomnia complaints (10.5%). Those with COMISA were referred for suspected SDB (77.8%) or insomnia complaints (22.2%). Referral indications did not differ significantly between the COMISA vs. OSA-only groups (*p* = 0.3). However, SDB tended to be a more frequent referral indication in the COMISA group than in the INS-only group (*p* = 0.05).

In the younger group, there were more type III OSA cases and fewer type I OSA cases in the OSA-only group than in the COMISA group. In the older group, the distribution of OSA types did not differ significantly between the COMISA and OSA-only groups.

#### 3.2.2. Polysomnographic Characteristics

##### Sleep Architecture

Sleep architecture did not differ between the COMISA and OSA-only groups in either age group.

In the older group, the arousal index was higher in both the COMISA and OSA-only groups compared to the INS-only group. Additionally, the proportion of N1 sleep and arousal-and-awakening index values were higher in the OSA-only group compared to the INS-only group.

##### Respiratory Parameters

In the younger group, respiratory parameters did not differ significantly between the COMISA and OSA-only groups. Although severe OSA was more common in the COMISA group and mild OSA more common in the OSA-only group, this difference was not statistically significant.

In the older group, OAHI values were higher in the OSA-only group compared to the COMISA group, although OSA was predominantly mild in both groups. The RERA index values were significantly higher in both the OSA-only and COMISA groups compared to the INS-only group. Additionally, ODI > 3 values were higher in the OSA-only group compared to the INS-only group.

#### 3.2.3. Questionnaires

**Figure 2:**
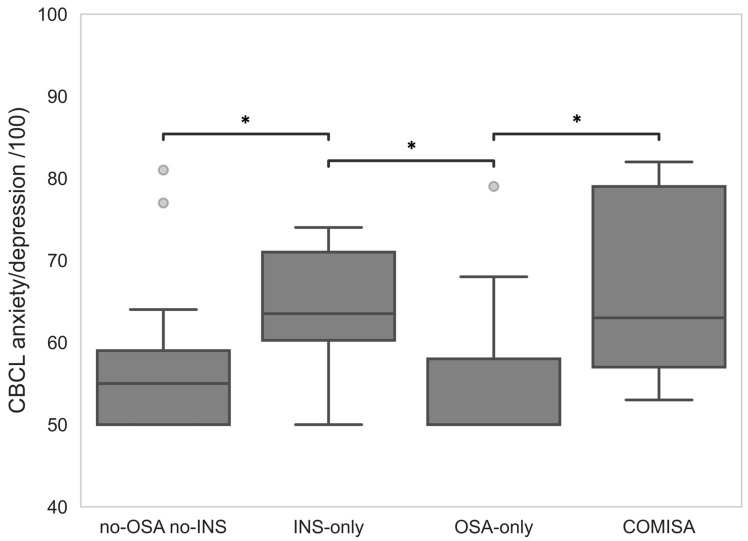
Child Behavior Checklist anxiety sub-scores (/100) per group for patients aged 4 years and older. Asterisks indicate significant differences between groups according to Mann–Whitney U tests (*p* < 0.05). Abbreviations: CBCL, Child Behavior Checklist; COMISA, comorbid insomnia and sleep apnea; INS, insomnia; OSA, obstructive sleep apnea.

In the older group, SDSC-DIMS scores were similar between the INS-only and COMISA groups, as were the levels of difficulties initiating and maintaining sleep. Daytime sleepiness was significantly greater in the OSA-only group compared to the INS-only group; however, no significant differences were found between COMISA and the other groups. CBCL-anxiety scores were higher in both the INS-only and the COMISA groups compared to the OSA-only group (Figure 2). CDI depression scores, Conners hyperactivity scores, and the proportion of school difficulties did not differ significantly across the four groups.

## 4. Discussion

This study evaluated the frequency and explored the characteristics of COMISA in children referred for PSG. COMISA was present in 14% of all patients and in 27% of those with confirmed OSA, with higher rates in younger children. Among the evaluated variables, only a lower OAHI and higher anxiety—both in older children—differentiated COMISA from isolated OSA.

The 14% overall frequency of COMISA observed in this study aligns with Meira e Cruz et al. [9] (16% in children aged 9–19 years), and exceeds the frequency reported by Yelov et al. [10] (8.7% in children aged 0.5–10 years). When focusing specifically on patients with confirmed OSA, the frequency of COMISA in this study (27%) was similar to that reported in previous studies (21% Meira e Cruz et al. [9], 18% in Yelov et al. [10]) and it remained substantially lower than in adults (39–55%) [6].

Consistent with Yelov et al. [10], COMISA was more frequent in younger children but showed no sex differences—unlike in adults, in whom female sex has been identified as a risk factor for COMISA [31]. As hypothesized by Yelov et al. [10], this may reflect hormonal influences on insomnia and, thus, on COMISA after puberty, though pubertal status was not assessed in the study herein.

Unlike in adults, the findings of the present study revealed no cumulative effect of OSA and insomnia in children. Insomnia frequency did not differ between children with and without OSA, consistent with a study by Calhoun et al., which reported similar rates of insomnia in children aged 5–10 years, regardless of the inclusion of patients with SDB [32]. Similarly, in this study, insomnia complaints were comparable in patients with and without OSA, whereas in adults, COMISA is more often associated with difficulties maintaining sleep [33,34]. The type of OSA may play a role in these differences. In the younger group, OSA was predominantly type I in children with COMISA and type III in those with isolated OSA. However, previous pediatric studies provide limited insight into the influence of OSA type on COMISA, as patients with complex comorbidities were either not included [10] or not specifically described [9]. This observed difference may, in part, reflect variation in referral patterns. Indeed, young patients with complex comorbidities are often screened for SDB regardless of the presence of sleep-related complaints, whereas children with type I OSA typically require more severe or noticeable symptoms to be referred for PSG. As a result, they may be more likely to present with complex sleep disorders such as COMISA.

In the older group, OAHI was lower in patients with COMISA compared to those with isolated OSA, aligning with a study by Krakow et al. [35] in which apnea-hypopnea index values were lower in adults with co-occurring SDB and insomnia compared to those with isolated SDB. Interestingly, in our sample, the difference in OSA severity was not accompanied by any other differences in PSG measures. Insomnia did not appear to worsen the objective sleep quality of children with OSA, contrasting with the findings in adults, in whom COMISA is typically associated with more pronounced sleep disruption [36]. Conversely, patients with COMISA exhibited a higher frequency of arousals compared to those with isolated insomnia but it did not significantly affect overall sleep architecture or daytime functioning like daytime sleepiness or hyperactivity.

In the present study, anxiety emerged as the only clinical feature differentiating children with COMISA from those with isolated OSA: Anxiety levels were higher in patients with insomnia, regardless of the presence of OSA. This is consistent with findings in the general pediatric population, where insomnia is commonly associated with anxiety [32]. In adults, COMISA is also linked to greater emotional distress, cognitive complaints, and reduced quality of life compared to isolated OSA [37]. These findings suggest that, in children, anxiety might be a key risk factor for the development of COMISA and should, therefore, be systematically assessed in patients with OSA.

The diverging findings between pediatric and adult COMISA likely reflect age-related changes in the pathophysiology of both OSA and insomnia, thus impacting the mechanisms underlying COMISA. Indeed, the pathophysiology of insomnia evolves across developmental stages, being mostly behavioral in early childhood and, rather, associated with poor sleep hygiene and phase delay in adolescence [3,32]. Concerning OSA, the first cause in childhood is adeno-tonsillar hypertrophy, likely due to the involvement of these tissues in immune mechanisms, which can be repeatedly triggered during childhood due to life in the community exposing them to frequent infectious episodes [38,39]. Later in adolescence, obesity becomes a more frequent cause of OSA and is one of its main risk factors in adulthood [40,41].

The main limitation of the present study is the relative small sample size, especially in the younger group, hindering the comparison of children with COMISA and those with isolated insomnia. Additionally, some questionnaires were not systematically administered, reducing the sample size available for certain retrospective analyses. Another limitation is the selective nature of the study population, as it was conducted in a specialized referral center; therefore, the findings may not be generalizable to the broader pediatric population.

The main strength of the study is the use of a standardized and consistent methodology across a broad pediatric age range, combined with objective diagnosis of OSA and detailed assessment of sleep architecture based on PSG.

In conclusion, this retrospective cross-sectional polysomnographic study demonstrated a lower frequency of COMISA in children compared to adults. No significant association between OSA and insomnia was found in children aged 6 months to 16 years. None of the polysomnographic or clinical characteristics examined were specific to COMISA. However, children with COMISA had a lower severity of OSA than those with isolated OSA, and they also exhibited higher anxiety levels. Therefore, it seems important to assess anxiety in pediatric OSA as it may contribute to the development of COMISA. For this purpose, the CBCL or shorter standardized questionnaires evaluating anxiety like the Hospital Anxiety and Depression Scale [42] could be easily integrated into the clinical practice in pediatric sleep medicine. The present findings suggest that, in children, insomnia and OSA may arise from distinct rather than mutually reinforcing mechanisms. However, caution is warranted when drawing conclusions given the exploratory nature of these findings. Future longitudinal studies should explore the impact of OSA management on the course of insomnia and further clarify the relationship between these two disorders in childhood.

## Figures and Tables

**Table 1:** Demographic characteristics, obstructive sleep apnea characterization, and insomnia scores according to age group.

	<4 Years Old (*n* = 46)	4–16 Years Old (*n* = 154)	*p*	Effect Size
General characteristics				
Age (years)	2.5 [0.6–3.9]	10.9 [4.1–16.7]	-	-
Sex				
Females	21 (45.7)	74 (48.1)	0.9	0.02
BMI z-score	0.4 [−2.7–5.5]	0.5 [−2.1–18.3], *n* = 153	0.2	0.1
Obesity	3 (10.3), *n* = 29	18 (11.8), *n* = 153	1	0.02
Pre-term	7 (15.2)	15 (10.6), *n* = 142	0.4	0.06
Neurodevelopmental disorder	9 (19.6)	65 (42.2)	0.005	0.2
ADHD	-	19 (12.3)	-	
Epilepsy	1 (2.2)	15 (9.7)	0.1	0.1
Treatments				
Wake-promoting only	0 (0.0)	15 (9.7)	0.03	0.2
Sleep-inducing only	2 (4.3)	17 (11.0)	0.3	0.1
Both	0 (0.0)	21 (13.6)	0.005	0.2
Reason for PSG				
SDB	41 (89.1)	88 (57.1)	0.003	0.3
Insomnia	2 (4.3)	39 (25.3)		
Hypersomnia	1 (2.2)	24 (15.6)		
Parasomnia	2 (4.3)	3 (1.9)		
OSA				
OAHI (/h)	5.0 [0.0–43.9]	1.6 [0.0–64.9]	<0.001	0.3
Pathological OAHI (≥2/h)	36 (78.3)	66 (42.9)	<0.001	0.5
Mild OSA [2–5)/h	13 (36.1)	36 (54.5)	0.1	0.2
Moderate OSA [5–10)/h	6 (16.7)	11 (16.7)		
Severe OSA ≥ 10/h	17 (47.2)	19 (28.8)		
Type I	17 (47.2)	26 (39.4)	0.2	0.2
Type II	0 (0)	5 (7.6)		
Type III	19 (52.8)	35 (53.0)		
Insomnia				
SDSC-DIMS	15.2 [8.0–34.0]	15.0 [7.0–31.0]	-	
Pathological insomnia	21 (45.7)	29 (18.8)	<0.001	0.3
SDSC-initiation/20	7.0 [4–19]	8.0 [4–19]	0.05	0.1
SDSC-maintaining/5	2.0 [1–5]	2.0 [1–5]	0.2	0.1
COMISA				
In all patients	18 (39.1)	9 (5.8)	<0.001	0.4
In OSA subgroup	18 (50.0)	9 (13.6)	<0.001	0.4

Data are expressed as median [range] or n (%). Abbreviations: ADHD, attention deficit hyperactivity disorder; BMI, body mass index; COMISA comorbid insomnia and sleep apnea; DIMS, Disorders of Initiating and Maintaining Sleep; OAHI, obstructive apnea-hypopnea index; OSA, obstructive sleep apnea; PSG, polysomnography; SDB, sleep disordered breathing; SDSC, Sleep Disturbance Scale for Children.

**Table 2:** General characteristics, polysomnographic data, and insomnia in patients with obstructive sleep apnea under 4 years old (*n* = 36).

	OSA-Only (*n* = 18)	COMISA (*n* = 18)	*p*	Effect Size
General characteristics				
Age (years)	2.5 [0.7–3.8]	2.4 [0.6–3.9]	0.9	0.02
Sex				
Females	10 (55.6)	7 (38.9)	0.5	0.2
BMI z-score	1.0 [−2.4–5.5]	0.7 [−2.2–2.5]	0.6	0.1
Obesity	3.0 (27.3), *n* = 11	0 (0), *n* = 11	0.2	0.4
Neurodevelopmental disorder	5 (27.8)	4 (22.2)	1	0.06
Epilepsy	0 (0)	1 (5.6)	1	0.2
Sleep-inducing treatment	1 (5.6)	1 (5.6)	1	0
OSA type				
Type I	4 (22.2)	13 (72.2)	0.008	0.5
Type III	14 (77.8)	5 (27.8)		
PSG sleep architecture				
TST (min)	497.5 [396.0–667.0]	526.0 [366.0–644.0]	0.3	0.2
SOL (min)	11.4 [1.3–405.5], *n* = 17	9.4 [2.4–194.0], *n* = 17	0.8	0.06
SE (%)	81.2 [63.4–96.8]	86.8 [68.1–96.0]	0.4	0.2
WASO (min)	107.2 [21.0–239.0]	83.0 [24.0–205.0]	0.4	0.2
N1 sleep (%TST)	11.6 [4.2–30.3]	15.3 [7.5–20.3]	0.4	0.2
N2 sleep (%TST)	0.4 [0.3–71.1]	0.4 [0.1–61.6]	0.9	0.02
N3 sleep (%TST)	25.4 [0.0–39.3]	27.3 [7.3–43.3]	1	0.01
REM sleep (%TST)	27.8 [18.9–35.6]	24.4 [16.5–35.1]	0.4	0.2
Arousal index (/h)	28.8 [6.2–72.0]	31.8 [8.7–44.5]	0.8	0.04
Arousal-and-awakening index (/h)	32.7 [12.5–86.5]	37.2 [12.0–52.5]	0.8	0.05
PLMI(/h)	6.1 [0.0–11.3], *n* = 9	3.3 [0.0–12.9], *n* = 11	0.2	0.3
PSG respiratory parameters				
OAHI (/h)	5.4 [2.0–29.4]	11.7 [2.7–43.9]	0.2	0.2
Mild OSA [2–5)/h	9 (50.0)	4 (22.2)	0.2	0.3
Moderate OSA [5–10)/h	3 (16.7)	3 (16.7)		
Severe OSA ≥ 10/h	6 (33.3)	11 (61.1)		
RERA (/h)	9.6 [0.2–20.2]	12.2 [2.6–18.3]	0.6	0.09
Mean SpO_2_ (%)	96.5 [94.6–98.3]	96.9 [92.1–98.5]	0.9	0.02
SpO_2_ < 90% (min)	0.6 [0.0–35.8]	0.6 [0.0–94.9]	1	0
ODI > 3 (/h)	7.5 [0.8–25.1]	7.8 [0.3–74.8]	0.4	0.1
Mean CO_2_ (mmHg)	40.1 [37.0–52.1], *n* = 17	42.1 [33.5–49.0]	0.9	0.02
CO_2_ > 50 mmHg (%)	0.0 [0.0–69.5], *n* = 17	0.0 [0.0–10.0], *n* = 17	0.8	0.04
Insomnia				
SDSC-DIMS/40	11.0 [9.0–16.0]	23.5 [17.0–34.0]	<0.001	0.9
SDSC-initiation/20	6.0 [4–8]	11.5 [5–19]	<0.001	0.7
SDSC-maintaining/5	1.0 [1–3]	3.8 [1–5]	<0.001	0.7

Data are expressed as median [range] or n (%). Abbreviations: BMI, body mass index; COMISA, comorbid insomnia and sleep apnea; DIMS, Disorders of Initiating and Maintaining Sleep; OAHI, obstructive apnea and hypopnea index; OSA, obstructive sleep apnea; PLMI, periodic leg movement index; REM, rapid eye movement; RERA, respiratory effort-related arousal; SDSC, Sleep Disturbance Scale for Children; SE, sleep efficacy; SOL, sleep onset latency; TST, total sleep time; WASO, wake after sleep onset; CO_2_ > 50 mmHg, time spent with CO_2_ values above 50 mmHg; ODI > 3, oxygen desaturation index > 3%; SpO_2_, oxygen saturation; PSG, polysomnography.

**Table 3:** General characteristics, polysomnographic data, questionnaires, and school difficulties in the older group (*n* = 154).

	no-OSA no-INS (*n* = 68)	INS-Only (*n* = 20)	OSA-Only (*n* = 57)	COMISA (*n* = 9)	*p*
General characteristics					
Age (years)	10.5 [4.6–16.1]	11.5 [5.5–16.7]	10.7 [4.1–16.6]	9.3 [4.1–16.7]	0.3
Sex					
Females	34 (50.0)	9 (45.0)	27 (47.4)	4 (44.4)	0.9
BMI z-score	0.0 [−1.9–9.0]	0.9 [−1.6–8.2]	1.2 [−2.1–18.3], *n* = 56	0.2 [−1.3–9.3]	0.4
Obesity	7 (10.3)	2 (10.0)	8 (14.3), *n* = 56	1 (11.1)	0.9
Neurodevelopmental disorder	30 (44.1)	9 (45.0)	22 (38.6)	4 (44.4)	0.9
ADHD	11 (16.2)	5 (25.0)	2 (3.5)	1 (11.1)	0.05 ^c+^
Epilepsy	7 (10.3)	1 (5.0)	6 (10.5)	1 (11.1)	0.9
Treatments					
Wake-promoting only	7 (10.3)	2 (10.0)	6 (10.5)	0 (0)	0.8
Sleep-inducing only	9 (13.2)	0 (0)	6 (10.5)	2 (22.2)	0.3
Both	10 (14.7)	4 (20.0)	5 (8.8)	2 (22.2)	0.5
OSA type					
Type I	-	-	21 (36.8)	5 (55.6)	0.4
Type II	-	-	4 (7.0)	1 (11.1)	
Type III	-	-	32 (56.1)	3 (33.3)	
PSG sleep architecture					
TST (min)	536.0 [273.0–665.0]	499.0 [395.0–610.0]	502.0 [264.0–684.0]	499.0 [411.0–548.0]	0.1
SOL (min)	19.8 [0.8–190.9]	26.8 [5.4–299.0]	14.8 [1.3–293.5], *n* = 56	21.9 [3.7–80.6]	0.1
SE (%)	89.9 [62.9–97.6]	91.2 [71.6–96.9]	86.7 [52.4–97.7]	88.6 [73.4–95.1]	0.1
WASO (min)	57.8 [14.0–217.0]	46.8 [16.5–193.0]	75.5 [13.5–256.5]	62.0 [28.5–180.5]	0.06
N1 sleep (%TST)	9.7 [2.3–23.3]	8.6 [0.9–15.6]	11.6 [2.0–32.8]	8.4 [4.7–14.7]	0.02 ^c+^
N2 sleep (%TST)	40.0 [0.3–56.7]	41.3 [0.3–60.0]	37.7 [0.3–69.8]	47.3 [0.4–60.4]	0.2
N3 sleep (%TST)	24.2 [1.0–40.6]	24.7 [9.7–41.1]	23.0 [0.6–37.1]	23.2 [7.1–34.0]	0.5
REM sleep (%TST)	22.8 [11.9–35.4]	21.0 [10.2–31.4]	22.2 [7.4–30.9]	22.5 [19.5–31.7]	0.7
Arousal index (/h)	15.0 [2.7–39.5]	9.9 [4.3–18.5]	22.4 [4.9–75.3]	16.8 [8.3–27.5]	<0.001 ^a#,c#^
Arousal-and-awakening index (/h)	21.3 [4.0–43.0], *n* = 39	17.8 [6.0–24.9], *n* = 8	27.2 [8.2–90.8], *n* = 50	22.0 [12.5–33.7], *n* = 6	0.006 ^c+^
PLMI (/h)	1.6 [0.0–22.5], *n* = 65	2.5 [0.0–15.8]	1.8 [0.0–27.8], *n* = 55	4.3 [0.0–12.5], *n* = 8	0.5
PSG respiratory parameters					
OAHI (/h)	0.7 [0.0–1.9]	0.4 [0.0–1.5]	4.7 [2.1–64.9]	3.3 [2.1–5.8]	<0.001 ^a#, b+, c#^
Mild OSA [2–5)/h	-	-	29 (50.9)	7 (77.8)	0.1
Moderate OSA [5–10)/h	-	-	9 (15.8)	2 (22.2)	
Severe OSA ≥ 10/h	-	-	19.0 (33.3)	0 (0)	
RERA (/h)	1.9 [0.2–9.5]	1.1 [0.1–6.7]	8.1 [0.7–45.4]	6.0 [1.6–8.9]	<0.001 ^a#,c#^
Mean SpO_2_ (%)	96.8 [94.7–98.3], *n* = 67	96.6 [92.4–97.7]	96.3 [91.9–98.7], *n* = 56	97.3 [93.1–97.8]	0.04
SpO_2_ < 90% (min)	0.0 [0.0–9.7], *n* = 67	0.0 [0.0–9.2]	0.0 [0.0–54.4], *n* = 56	0.0 [0.0–0.0]	0.09
ODI > 3 (/h)	2.1 [0.0–11.2], *n* = 67	1.4 [0.0–8.0]	4.2 [0.2–62.7], *n* = 56	2.4 [0.4–9.2]	<0.001 ^c+^
Mean CO_2_ (mmHg)	42.8 [36.4–48.8], *n* = 66	42.6 [34.6–49.2]	42.7 [33.2–49.1], *n* = 56	42.4 [38.7–50.4]	0.7
CO_2_ > 50 mmHg (%)	0.0 [0.0–35.4], *n* = 66	0.0 [0.0–47.7]	0.0 [0.0–45.1], *n* = 56	0.0 [0.0–67.8]	0.5
Questionnaires					
SDSC-DIMS/35	13.0 [7–21]	25.5 [22–31]	13.0 [2–21]	26.0 [22–31]	<0.001 ^b#,c#^
SDSC-initiation/20	-	15.5 [12–19]	-	17.0 [13–18]	0.9
SDSC-maintaining/5	-	3.0 [2–5]	-	4.0 [2–5]	0.3
FSSA/24	6.0 [0–24], *n* = 61	3.5 [0–10], *n* = 18	6.0 [0–21], *n* = 46	6.5 [1–11]	0.06
FSSA > 10	11 (18.0), *n* = 61	0 (0), *n* = 18	16 (37.2), *n* = 46	1 (12.5), *n* = 8	0.007 ^c+^
CDI/54	10.0 [2–23], *n* = 34	13.5 [6–39], *n* = 12	14.5 [4–28], *n* = 10	17.0 [8–24], *n* = 3	0.1
CDI ≥ 16	9 (26.5), *n* = 34	5 (41.7), *n* = 12	5 (50.0), *n* = 10	2 (66.7), *n* = 3	0.3
CBCL anxiety/depression/100	55.0 [50–81], *n* = 29	63.5 [50–74], *n* = 8	50.0 [50–79], *n* = 29	63.0 [53–82], *n* = 5	0.008 ^b+,c+^
CBCL ≥ 70	2 (6.9), *n* = 29	3 (37.5), *n* = 8	1 (3.4), *n* = 29	2 (40.0), *n* = 5	0.008
Conners/30	10.0 [0–26], *n* = 57	14.0 [2–27], *n* = 16	11.0 [0–27], *n* = 38	14.0 [4–22]	0.5
Conners > 15	13 (22.8), *n* = 57	7 (43.8), *n* = 16	11 (28.9), *n* = 38	4 (50.0)	0.2
School difficulties	25 (41.0), *n* = 61	4 (23.5), *n* = 17	21 (45.7), *n* = 46	3 (33.3)	0.4

Data are expressed as median [range] or n (%). Exponent letters indicate significant post hoc Mann–Whitney or Fisher’s exact test: (^a^) INS-only vs. COMISA, (^b^) OSA-only vs. COMISA, (^c^) INS-only vs. OSA-only. Symbols indicate medium (^+^) and large (^#^) effect size. Abbreviations: ADHD, attention deficit hyperactivity disorder; BMI, body mass index; CBCL, Child Behavior Checklist; CDI, Children’s Depression Inventory; COMISA, comorbid insomnia and sleep apnea; DIMS, Disorders of Initiating and Maintaining Sleep; FSSA, French sleepiness scale for adolescents; INS, insomnia; N1/N2/N3, sleep stages 1/2/3; OAHI, obstructive apnea and hypopnea index; ODI > 3, oxygen desaturation index > 3%; OSA, obstructive sleep apnea; PLMI, periodic leg movement index; PSG, polysomnography; REM, rapid eye movement; RERA, respiratory effort-related arousal; SDSC, Sleep Disturbance Scale for Children; SE, sleep efficacy; SOL, sleep onset latency; TST, total sleep time; WASO, wake after sleep onset.

## Data Availability

Data are not available due to ethical and privacy restrictions. Data may be available on request from the corresponding author and after additional and documented consent of parents involved in this study.

## Abbreviations

The following abbreviations are used in this manuscript:

AASM	American Academy of Sleep Medicine
ADHD	Attention Deficit Hyperactivity Disorder
BMI	Body Mass Index
CAHI	Central Apnea-Hypopnea Index
CBCL	Child Behavior Checklist
CDI	Children’s Depression Inventory
COMISA	COMorbid Insomnia and Sleep Apnea
CPAP	Continuous Positive Airway Pressure
DIMS	Disorders of Initiating and Maintaining Sleep
EEG	Electroencephalogram
FDR	False Discovery Rate
FSSA	French Sleepiness Scale for Adolescents
GA	Gestational Age
OAHI	Obstructive Apnea-Hypopnea Index
ODI > 3	Oxygen Desaturation Index >3%
OSA	Obstructive Sleep Apnea
PLMI	Periodic Leg Movement Index
PSG	Polysomnography
REM	Rapid Eye Movement
RERA	Respiratory Effort-Related Arousal
SDB	Sleep Disordered Breathing
SDSC	Sleep Disturbance Scale for Children
SE	Sleep Efficacy
SOL	Sleep Onset Latency
SpO_2_	Oxygen Saturation
TST	Total Sleep Time
WASO	Wake After Sleep Onset
